# Associations Between Cerebral Perfusion Pressure, Hemodynamic Parameters, and Cognitive Test Values in Normal-Tension Glaucoma Patients, Alzheimer’s Disease Patients, and Healthy Controls

**DOI:** 10.3390/medicina61060972

**Published:** 2025-05-24

**Authors:** Akvile Stoskuviene, Edvinas Chaleckas, Evelina Grusauskiene, Laimonas Bartusis, Guven Celikkaya, Ingrida Januleviciene, Antanas Vaitkus, Arminas Ragauskas, Yasin Hamarat

**Affiliations:** 1 Department of Neurology, Medical Academy, Lithuanian University of Health Sciences, LT-44307 Kaunas, Lithuania; akvile.stoskuviene@lsmu.lt (A.S.); antanas.vaitkus@lsmu.lt (A.V.); 2 Eye Clinic, Lithuanian University of Health Sciences, Eiveniu Str. 2, LT-50161 Kaunas, Lithuania; ingrida.januleviciene@kaunoklinikos.lt; 3 Health Telematics Science Institute, Kaunas University of Technology, LT-51423 Kaunas, Lithuania; edvinas.chaleckas@ktu.lt (E.C.); laimonas.bartusis@ktu.lt (L.B.); arminas.ragauskas@ktu.lt (A.R.); 4 Visual Communication Design, Faculty of Art, Design and Architecture, Isik University, Mesrutiyet Mah., Universite Sk., No:2, Sile, TR-34980 Istanbul, Turkey; 5 Laboratory of Heat-Equipment Research and Testing, Lithuanian Energy Institute, Breslaujos Str. 3, LT-44403 Kaunas, Lithuania

**Keywords:** normal tension glaucoma, Alzheimer’s disease, cognitive test, cerebral perfusion pressure

## Abstract

*Background/Objectives*: Glaucoma and Alzheimer’s disease (AD) are neurodegenerative conditions with vascular underpinnings. This study aimed to explore the relationship between blood pressure parameters such as mean arterial pressure (MAP), pulse pressure (PP), and cerebral perfusion pressure (CPP) and cognitive performance in patients with AD, normal-tension glaucoma (NTG), and healthy controls. We hypothesized that NTG patients, like those with mild cognitive impairment (MCI), may experience subtle cognitive changes related to vascular dysregulation. *Methods*: Ninety-eight participants (35 NTG, 17 AD, 46 controls) were assessed for CPP, MAP, OPP, and cognitive performance. Statistical analyses compared groups and examined correlations. *Results*: AD patients showed lower CPP and MAP (*p* < 0.001), indicating systemic vascular dysfunction, while NTG patients had higher ocular perfusion pressure (OPP) (*p* = 0.008), suggesting compensatory mechanisms. CPP correlated with visuospatial abilities in AD (r = 0.492, *p* = 0.045). MAP correlated with the Clock drawing test (CDT) scores in the NTG group (r = 0.378, *p* = 0.025). PP negatively correlated with cognition in AD (r = −0.527, *p* = 0.016 for CDT scores) and controls (r = −0.440, *p* = 0.002 for verbal fluency and r = −0.348, *p* = 0.019 for total ACE scores). *Conclusions*: The study highlights distinct hemodynamic profiles: systemic dysfunction in AD and localized dysregulation in NTG. These findings emphasize the role of vascular dysregulation in neurodegeneration, with implications for personalized treatment approaches targeting vascular health in neurodegenerative conditions.

## 1. Introduction

Glaucoma is a group of ocular disorders characterized by progressive damage to the optic nerve, leading to irreversible vision loss. It is one of the leading causes of irreversible blindness worldwide, affecting over 70 million people [1,2]. The most common form, primary open-angle glaucoma (POAG), is often associated with elevated intraocular pressure (IOP). However, a significant subset of glaucoma patients, particularly those with normal-tension glaucoma (NTG), exhibit optic nerve damage despite IOP within the normal range [3,4].

NTG is a subtype of glaucoma where optic nerve damage and visual field loss occur without elevated IOP. It accounts for approximately 20–30% of all glaucoma cases, with a higher prevalence in Asian populations [5]. The pathogenesis of NTG is not fully understood, but is thought to involve vascular dysregulation, oxidative stress, and neurodegenerative processes [4,5,6,7]. Unlike high-tension glaucoma, NTG is often associated with systemic conditions such as migraines, Raynaud’s phenomenon, and low blood pressure, suggesting a broader systemic involvement [4,5,7].

Alzheimer’s Disease (AD) is a progressive neurodegenerative disorder characterized by the accumulation of amyloid-beta plaques and tau tangles in the brain, leading to memory loss, cognitive decline, and behavioral changes [8]. Mild Cognitive Impairment (MCI) is a transitional stage between normal aging and dementia, characterized by noticeable cognitive decline that does not significantly interfere with daily activities [9]. MCI is a significant risk factor for Alzheimer’s Disease (AD), with approximately 10–15% of MCI patients progressing to AD annually [10].

Arterial blood pressure (ABP) dysregulation is common in AD and NTG patients. In AD, systemic hypotension, particularly nocturnal hypotension, has been associated with reduced cerebral perfusion, exacerbating neurodegeneration [11]. Similarly, NTG patients often exhibit lower systemic ABP, which may contribute to insufficient ocular perfusion and optic nerve damage [7]. This shared vascular dysregulation suggests that systemic hypotension may be a risk factor for both conditions, linking vascular health to neurodegeneration in the brain and the eye.

Emerging evidence suggests a potential link between glaucoma and AD. Studies have shown that glaucoma patients, particularly those with NTG, have a higher prevalence of MCI and AD compared to the general population [12]. For example, a population-based study found that glaucoma patients were 1.5 times more likely to develop AD than age-matched controls [13]. This association raises the possibility that glaucoma, particularly NTG, may share common neurodegenerative pathways with AD.

This study aimed to explore the relationship between blood pressure parameters, such as mean arterial pressure (MAP), pulse pressure (PP), cerebral perfusion pressure (CPP), and cognitive performance in patients with AD, NTG, and healthy controls. We hypothesized that NTG patients, like those with MCI, may experience subtle cognitive changes related to vascular dysregulation. By investigating these relationships, the study seeks to understand better how vascular health influences cognitive decline in NTG and AD, to inform personalized treatment approaches.

## 2. Materials and Methods

### 2.1. Study Design and Participants

The study was conducted at the Hospital of the Lithuanian University of Health Sciences Kaunas Clinics, with participant recruitment and assessments occurring between May 2017 and November 2018. Post hoc analysis and interpretation of the data were conducted from October 2024 to February 2025. Ethical approval was obtained from the Kaunas Regional Biomedical Research Ethics Committee (Approval No. BE-2-28, dated 5 May 2017), and the study adhered to the principles of the Declaration of Helsinki. A total of 100 participants were initially enrolled, but after applying the exclusion criteria, 2 participants were excluded: 1 from the NTG group and 1 from the Control group, resulting in 98 participants older than 65 years were included and categorized into three groups: the NTG group (*n* = 35), consisting of patients diagnosed with NTG based on optic nerve head changes, visual field defects, open anterior chamber angles, and IOP ≤ 21 mmHg; the AD group (*n* = 17), comprising patients with diagnosed mild to moderate probable AD according to NINCDS-ADRDA Alzheimer’s criteria; and the control (C) group (*n* = 46), which included cognitively healthy individuals without glaucoma or neurodegenerative disorder confirmed after ophthalmologist and neurologist evaluation.

The groups were matched for age and anthropometric parameters. Group-level matching was applied based on age, sex, and body mass index (BMI), ensuring no statistically significant differences in these parameters between study groups. Participants were excluded if they met the following criteria:

- Age under 65 or over 85 years.
- History of ocular trauma or previous ocular surgery, refractive error greater than three diopters.
- Severe and uncompensated systemic conditions that could influence study outcomes (e.g., decompensated cardiovascular disease, acute or chronic respiratory disease, diabetes mellitus, severe renal or hepatic dysfunction, or active cancer).
- Prominent neurological deficit (paresis, ataxia, aphasia, etc.), evident extrapyramidal signs (tremor, rigidity) or psychiatric disorders (severe depression, psychotic type).
- Allergy to local anesthetics.

### 2.2. Clinical and Cognitive Assessments

All participants underwent a comprehensive ophthalmologic examination, including best-corrected visual acuity (BCVA), spherical equivalent (SE), and IOP measurements (Goldmann applanation tonometry, Haag-Streit, Koniz, Switzerland). Visual field was assessed (24-2 SITA-Fast strategy; Humphrey Standard Perimetry, Carl Zeiss Meditec, Germany). In addition, ocular hemodynamic parameters such as ocular perfusion pressure (OPP), systolic perfusion pressure (SPP), diastolic perfusion pressure (DPP), translaminar pressure gradient (TLPG, calculated as IOP—intracranial pressure (ICP)), and cerebral perfusion pressure (CPP, calculated as MAP—ICP) were recorded to assess vascular dynamics relevant to glaucoma and neurodegenerative conditions. A detailed medical anamnesis was collected.

Three blood pressure (BP) measurements were performed manually on the right arm following standard clinical guidelines. To ensure consistency, all measurements were taken in a temperature-controlled environment (22–24 °C) during the morning (9:00–10:00 a.m.). Participants were advised to refrain from consuming caffeine, alcohol, or smoking for at least 12 h before the assessment. The average of the second and third BP readings was used for analysis to minimize variability. MAP was calculated as diastolic BP (DBP) + (PP/3), where PP was determined as the difference between systolic BP (SBP) and DBP. CPP was then derived using the formula MAP—ICP.

Noninvasive ICP was measured using a two-depth transcranial Doppler (TCD) device, which does not require patient-specific calibration. This method is based on the simultaneous monitoring of blood flow velocity pulsations in the ophthalmic artery’s intracranial and extracranial segments. Patients remained supine with their eyelids closed, while a special head frame with a fixed ultrasound transducer was placed over the closed eyelid. A special acoustic gel was applied to improve ultrasonic contact. External pressure (Pe) was generated using a small ring cuff placed over the tissues surrounding the eyeball. Pe was automatically increased in gradual steps of 4 mmHg from 0 to 20 mmHg. If the initial ICP measurement was lower than 10 mmHg, additional measurements were performed with Pe increased by 2 mmHg increments up to 12 mmHg to minimize sampling error. The entire procedure lasted up to 10 min [14].

To evaluate cognitive function, participants completed neuropsychological tests, including the Clock Drawing Test (CDT), the Addenbrooke’s Cognitive Examination (ACE-RLT), and the Mini-Mental State Examination (MMSE) as a subsection of the ACE-RLT test. Subsections of ACE-RLT assessments targeted key cognitive domains such as attention, visuospatial abilities, and executive function. Although CDT is included in the ACE-RLT and MMSE, we have also included a different CDT version. The predrawn circle of 10 cm diameter was given, and patients were asked to draw the clock at 11:10. Such a time includes both visual fields and highlights patients who tended to be “pulled” (frontal pull due to executive dysfunction) while setting this time. There is no time limit to perform the test. Usually, it takes about 2 min. CDT was assessed using the 4-point Rakusa methodology [15]. These tests were selected to explore potential associations between cerebral and ocular perfusion parameters and cognitive performance, particularly in individuals with NTG and AD.

### 2.3. Statistical Analysis

All statistical analyses were conducted using IBM SPSS Statistics (version 30.0, IBM Corporation, Armonk, NY, USA). The Kolmogorov–Smirnov test was applied to evaluate the normality of continuous variables in all patients.

For variables that followed a normal distribution, such as age, CPP, MAP, and ICP, data were expressed as mean (standard deviation, SD), and group comparisons were performed using Student’s *t*-test. Non-normally distributed variables, including body mass index (BMI), SBP, and DBP, were presented as median [interquartile range, IQR] and analyzed using the Mann–Whitney U test.

Categorical data, such as gender distribution, were compared across groups using the Chi-square test. For multiple-group comparisons between NTG, AD, and C groups, one-way ANOVA was used. The Mann–Whitney U test and one-way ANOVA were followed by post hoc Bonferroni correction to adjust for multiple comparisons.

Pearson’s and Spearman’s correlation coefficients were calculated to explore relationships between blood pressure parameters (MAP, PP), CPP, and cognitive performance (CDT; scores of MMSE and ACE-RLT). A *p*-value of <0.05 was considered statistically significant for all analyses.

## 3. Results

A total of 98 participants were included in the study, divided into three groups: NTG group (n = 35), AD group (n = 17), and C group (n = 46). The baseline demographic and clinical characteristics of the study participants are presented in Table 1. The mean age did not differ significantly between groups (*p* = 0.169). Gender distribution also showed no significant difference (*p* = 0.135), and body mass index (BMI) was comparable across all groups (*p* = 0.843), ensuring a homogeneous study sample. Although the proportion of males in the AD group (47.1%) is more than twice as high as in the NTG group (21.6%) and nearly twice as high as in the control group (26.1%), this difference was not statistically significant (*p* = 0.135, χ^2^ test).

**Figure 1:**
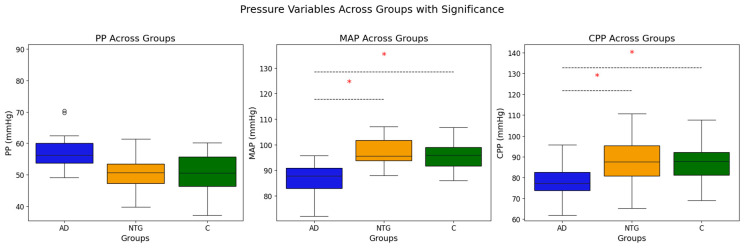
Boxplots of Pulse Pressure (PP), Mean Arterial Pressure (MAP), and Cerebral Perfusion Pressure (CPP) across study groups. C: Control group; AD: Alzheimer’s disease patients; NTG: Normal Tension Glaucoma patients. O indicate statistical outliers, * indicate statistical significant.

Blood pressure parameters revealed that AD patients had significantly lower SBP (*p* = 0.049), DBP (*p* < 0.001), and MAP (*p* = 0.001) compared to NTG and C groups, suggesting altered vascular regulation in AD. Additionally, CPP was significantly lower in AD patients compared to NTG and controls (*p* < 0.001), indicating potential cerebral hypoperfusion in AD. Boxplots of MAP, CPP, and PP are shown in Figure 1.

No significant differences were observed in IOP among groups. Visual function parameters, including best corrected visual acuity BCVA, mean deviation (MD), pattern standard deviation (PSD), and visual field index (VFI), were significantly worse in AD and NTG groups compared to C (*p* < 0.001). Notably, the AD group exhibited worse BCVA (*p* < 0.001) and VFI (*p* < 0.001), suggesting an association between neurodegeneration and visual impairment.

The study highlights systemic and ocular vascular dysregulation in AD, with notable differences in blood pressure and cerebral and ocular perfusion pressure parameters compared to NTG and controls. These findings suggest a potential link between neurodegeneration and impaired vascular function, warranting further research on the shared pathophysiological mechanisms of AD and NTG.

The analysis of cognitive test performance revealed significant correlations between CPP and cognitive function in the AD group (Table 2), specifically in visuospatial abilities (r = 0.492, *p* = 0.045), supporting decreased cerebral perfusion for cognitive abilities.

Table 3 displays that the NTG group differed from other groups with a significant positive correlation between MAP and the CDT (r = 0.378, *p* = 0.025). No significant relationships were observed with other cognitive test parts in the NTG group. There were no significant correlations between MAP and cognitive test scores, neither in the AD or C groups. These results suggest that MAP may influence cognitive function in NTG patients, but not in AD or C groups.

Significant correlations between PP and cognitive function were also determined, specifically in the AD group (r = −0.527, *p* = 0.016 for CDT) and the C group (r = −0.440, *p* = 0.002 for verbal fluency and r = −0.348, *p* = 0.019 for total ACE scores) (Table 4). However, no significant correlations were found in NTG patients. These findings suggest that systemic hemodynamics, particularly PP, may have varying implications for cognitive decline in different groups (Table 4).

**Figure 2:**
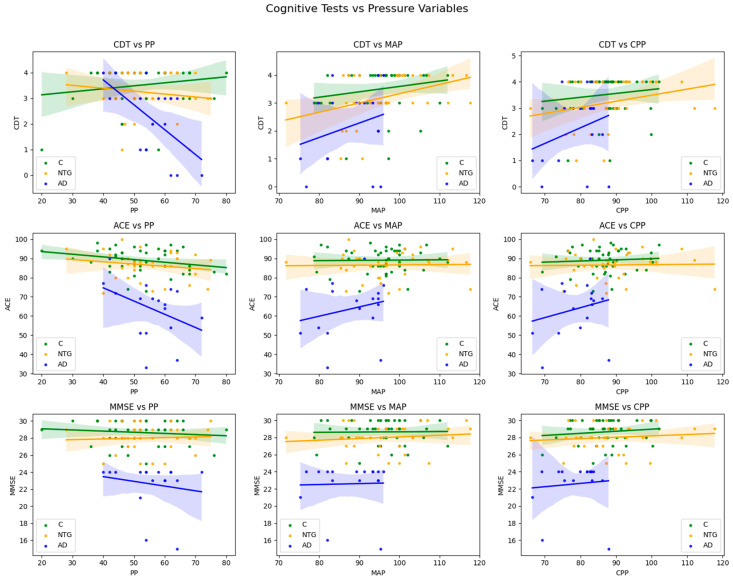
Relationship Between Blood Pressure Parameters, CPP, and Cognitive Function in NTG, AD, and C Groups. CDT: Clock Drawing Test; ACE: Addenbrooke’s Cognitive Examination; MMSE: Mini-Mental State Examination; PP: Pulse Pressure; MAP: Mean Arterial Pressure; CPP: Cerebral Perfusion Pressure. C: Control group; AD: Alzheimer’s disease patients; NTG: Normal Tension Glaucoma patients. Pearson’s correlation coefficient (r) was used for all statistical analyses.

Scatter plots (Figure 2) show the relationship between PP, MAP, and CPP and cognitive test scores in NTG, AD, and C groups. Each point represents an individual participant, and different marker styles indicate cognitive test types (CDT, ACE-RLT, and MMSE scores).

The plots revealed a narrower interval for all pressure parameters in the AD patient group. The correlation lines were steeper in the AD group. However, most correlations did not reach statistical significance, except a significant negative correlation between CDT and PP (r = −0.526, *p* = 0.03). The PP range was narrower, while the MAP and CPP values intervals were wider in the NTG group than in the C group. The correlation lines appeared to have a similar slope in both the NTG and C groups. However, PP displayed significant correlations with cognitive test results in the AD and C groups. In contrast, significant correlations were found between MAP values and cognitive test results in the NTG group. Such a distribution might suggest that NTG is more closely related to the C group than the AD group, based on hemodynamic parameters such as PP, MAP, and CPP.

## 4. Discussion

The findings of this study highlight significant differences in CPP, MAP, PP, and ocular perfusion pressure parameters among AD, NTG, and C groups, offering insights into the vascular and hemodynamic underpinnings of these neurodegenerative and glaucomatous conditions. Below, we discuss the implications of these findings in the context of the existing literature and propose potential mechanisms linking vascular dysregulation, neurodegeneration, and cognitive decline.

### 4.1. Hemodynamic Differences and Their Implications

The study revealed significant differences in CPP and MAP between the AD and NTG groups, with AD patients exhibiting lower CPP and MAP than NTG patients and controls. It is established that arterial hypertension is an essential factor in the development of the brain, in terms of both morphological and functional changes, such as hypertrophic vascular remodeling and cerebral hypoperfusion [16]. Reduced CPP in AD patients may reflect impaired cerebral blood flow, which has been implicated in the pathogenesis of AD [17].

The lack of significant differences in CPP between the NTG and controls is consistent with the hypothesis that NTG is primarily a localized optic neuropathy rather than a systemic vascular disorder [5]. The significantly higher OPP in NTG patients compared to AD patients underscores the importance of ocular hemodynamics in glaucoma pathogenesis. This observation supports the notion that impaired ocular blood flow contributes to glaucomatous optic neuropathy, as proposed by Flammer et al. [4].

Regarding hemodynamic ocular parameters, OPP, SPP, and DPP were significantly lower in AD patients compared to NTG and controls (*p* = 0.008, *p* = 0.02, and *p* = 0.024, respectively), indicating impaired ocular blood flow regulation in AD and reinforcing the hypothesis of vascular dysfunction in AD. Whereas non-invasive ICP was lower and TPG was higher in the NTG group compared to AD and healthy controls, implicating the idea that ICP plays a role in the pathogenesis of NTG [18].

### 4.2. Cognitive Function and Hemodynamic Correlations

The correlation analysis between MAP, CPP, PP, and cognitive test performance across the three study groups revealed mostly non-significant associations, with few notable exceptions. A positive correlation was found between CPP and visuospatial abilities sub-test scores (r = 0.492, *p* = 0.045) in AD patients. This finding aligns with the previous statements that CPP is an essential factor in AD [16]. Also, the AD group showed a significant correlation between CDT scores and PP (r = −0.527, *p* = 0.016). However, no significant correlations were found with other cognitive function domains despite these patients having lower CPP, MAP, and higher PP values overall. This result may be attributed to neurovascular uncoupling, a process in which cerebrovascular regulation becomes impaired, diminishing the impact of blood pressure on cognitive function as the disease progresses [17]. Additionally, AD patients in this study may have been in later disease stages, where structural brain degeneration outweighs the influence of hemodynamic factors. Another possible explanation for the lack of correlation in AD is the impact of cholinergic treatments, such as Donepezil, which could be modulating cognitive function independently of perfusion parameters [19]. This highlights the complexity of AD pathophysiology, where multiple overlapping mechanisms, including vascular, neurodegenerative, and pharmacological, may obscure the relationship between hemodynamics and cognition.

The multiplicity of processes related to the occurrence of AD could help explain the narrower intervals of PP, MAP, and CPP values in AD patients compared to the other groups. It is established that hypertension affects brain structure and function; however, this impact is complex and depends on other closely related factors such as age, the chronicity of hypertension, and antihypertensive treatment as well [16]. The variation in blood pressure parameters and CPP observed in our study might be due to disease-related vascular changes, autonomic dysfunction [11], or the homogeneous health conditions of AD patients. Moreover, lower MAP values have been associated with poorer cognitive performance in individuals with MCI, which could further explain the observed trends [9]. However, we did not assess the cognitive function changes according to the use of antihypertensive medications or the general health status of the participants in this study. The small size of the AD group could also influence such distribution.

In the C group, PP correlated with verbal fluency (r = −0.440, *p* = 0.002) and total ACE scores (r = −0.348, *p* = 0.019). The impact of PP on cognitive function remains ambiguous [20,21]. A recent study (performed by Mizuhara et al.) concluded that high PP was associated with cognitive function decline in subjects without dementia [22]. Contrarily, meta-analysis data revealed no associations between PP and the risk of developing cognitive function impairment [23]. Authors hypothesized that a neutral relationship could be justified according to the previously described U-shaped relationship between the value of PP and the prevalence of dementia, as both higher and lower tertiles of PP were related to an increased risk of the disease [24]. When defining this relationship, more factors have to be considered: SBP and DBP alterations in midlife and late-life; the associations with other BP-related factors, such as prehypertension, BP variability, BP reduction, orthostatic hypotension; and even AD-related factors as APOE allele that might modify the influence of BP values and cognitive decline [23].

Interestingly, MAP showed significant correlation with CDT scores in the NTG group (r = 0.378, *p* = 0.025) but no significant correlations with cognitive domains in the AD or C groups. This suggests that MAP alone may not be an adequate predictor of cognitive decline, at least in the context of this study population. While MAP is a key determinant of cerebral blood flow, it is essential to recognize that cerebral autoregulatory mechanisms work to maintain relatively stable perfusion, even when systemic MAP fluctuates [11]. This may explain why no direct associations between MAP and cognitive function were found. However, it is also possible that other factors, such as vascular resistance, blood–brain barrier integrity, and metabolic demand, play a more significant role in modulating the relationship between blood pressure and cognitive performance.

However, significant correlations were observed between CDT and hemodynamic parameters (MAP correlated with CDT results in the NTG group, whereas CDT correlated with PP in the AD group). This highlights that visuospatial function, which a simple and quick CDT can evaluate, depends on hemodynamic parameters and is affected in NTG patients. Consequently, traditionally identified as a disease limited to the eye, lately, more often, glaucoma is being considered a neurodegenerative disorder [25].

### 4.3. Ocular Perfusion and Glaucoma

The study found significant differences in OPP, SPP, and DPP among the groups, with AD patients exhibiting lower perfusion pressures than NTG patients and controls. Previous studies highlighted the role of ocular blood flow in glaucoma pathogenesis [4,26]. The higher OPP in NTG patients may reflect a compensatory response to maintain retinal and optic nerve head perfusion in the face of elevated IOP or vascular dysregulation.

Whereas we have selected NTG as the glaucoma group with IOP within normal limits, and, as expected, IOP did not differ between groups. Consequently, this supports the hypothesis that IOP-independent mechanisms, such as vascular dysregulation and oxidative stress, may play a more prominent role in NTG pathogenesis [6]. Furthermore, ICP was lower, and TPG was higher in the NTG group than in the other two study groups (however, results have not reached a significant level). These findings align with the growing recognition that NTG is a multifactorial disease involving both IOP-dependent and IOP-independent mechanisms [5,14,27].

### 4.4. Ocular Perfusion and AD

Altered ocular perfusion parameters determined by optical coherence tomography angiography were detected in the AD patient group compared with healthy controls. Authors linked these changes not specifically to AD pathology but to the vascular cerebral changes in AD [28].

### 4.5. Shared Mechanisms and Pathophysiological Overlaps

The findings of this study suggest that AD and NTG share some common pathophysiological features, particularly in terms of vascular dysregulation and oxidative stress. Both conditions are associated with impaired blood flow and increased oxidative stress, which contribute to neurodegeneration [6,29]. However, the distinct hemodynamic profiles observed in AD and NTG patients suggest that these conditions may involve different vascular pathways.

We have explored vascular dysregulation as a potential common pathogenetic pathway shared between AD and NTG. In addition to vascular factors, TLPG has been implicated in optic nerve damage, particularly in NTG. An increased TLPG can place mechanical stress on the lamina cribrosa, contributing to retinal ganglion cell loss. Moreover, as highlighted by Ho et al. [30], other overlapping mechanisms such as impaired CSF dynamics and accumulation of toxic substances manifesting as dysfunction of glymphatic system in the eye and brain contributing to neurodegeneration, as well as neuroinflammation that may also play a role in both conditions, reinforcing the concept of shared neurodegenerative pathways.

For example, the lower CPP and MAP in AD patients may reflect systemic vascular dysfunction, whereas the higher OPP in NTG patients may indicate localized vascular dysregulation in the eye. These differences may explain why AD is primarily associated with cognitive decline, while NTG is associated mainly with visual field loss. Nonetheless, the overlapping mechanisms suggest that therapeutic strategies targeting vascular dysregulation and oxidative stress may benefit both conditions.

While vascular dysregulation remains a central focus of this study, growing evidence highlights its intersection with neuroinflammatory processes in both Alzheimer’s disease (AD) and glaucoma. Retinal microvascular dysfunction has been demonstrated as an early feature shared between AD and primary open-angle glaucoma (POAG), suggesting common vascular insults may initiate neurodegeneration [31,32]. In AD, activated microglia drive neurovascular unit impairment through sustained neuroinflammation and oxidative stress [33], while in glaucoma models, microglial activation in the optic nerve head contributes to retinal ganglion cell death [34]. Notably, glymphatic system impairment at the lamina cribrosa has been proposed as a potential mechanism linking vascular and inflammatory pathology in glaucoma [30,35,36]. These parallel findings suggest that vascular dysfunction and neuroinflammation may synergistically accelerate disease progression across neurodegenerative conditions. Future research should investigate this vascular-inflammatory axis, particularly focusing on early biomarkers and therapeutic targets [37].

### 4.6. Limitations and Future Directions

While this study provides valuable insights into AD and NTG’s hemodynamic and cognitive correlations, several limitations should be acknowledged. First, the cross-sectional design limits our ability to establish causal relationships between hemodynamic parameters and disease progression. Also, we have not assessed the impact of using antihypertensive medications on cognitive function changes or general health status changes. Longitudinal studies are needed to determine whether changes in CPP, MAP, PP, and OPP precede or result from neurodegeneration.

The small sample size, particularly in the AD group, may have limited the statistical power to detect subtle differences between groups. As this is a preliminary study, further investigation with larger cohorts and more robust methodologies is necessary to confirm these findings, explore potential subgroup differences, and better understand the underlying mechanisms involved.

Third, for ICP measurement, we used a noninvasive two-depth transcranial Doppler (TCD) device instead of the “gold standard” invasive cerebrospinal fluid (CSF) column height measurement. We used this non-invasively evaluated ICP value in CPP and TPG formulas. The sensitivity, specificity, and diagnostic value of the TCD method were proven earlier [30,31].

Finally, the study did not assess other potential confounding factors, such as genetic predisposition, lifestyle factors, and comorbidities, which may influence hemodynamics and disease progression. Future studies should incorporate these factors to provide a more comprehensive understanding of the complex interplay between vascular dysregulation and neurodegeneration.

## 5. Conclusions

In conclusion, this study highlights the importance of systemic and ocular hemodynamics in the pathogenesis of AD and NTG. The findings suggest that AD is associated with systemic vascular dysfunction, as evidenced by reduced CPP and MAP. In contrast, NTG is related to localized vascular dysregulation in the eye, as evidenced by elevated OPP. These differences may explain the distinct clinical manifestations of these conditions and underscore the need for targeted therapeutic strategies.

The significant correlation between CDT and MAP in the NTG group (r = 0.378, *p* = 0.025) emphasizes the potential role of hemodynamic disturbances in cognitive performance, even in a condition primarily characterized by optic nerve degeneration. PP negatively correlated with cognition in AD (r = −0.527, *p* = 0.016 for CDT scores) and controls (r = −0.440, *p* = 0.002 for verbal fluency and r = −0.348, *p* = 0.019 for total ACE scores), highlighting the importance of hemodynamic disturbances for stable cognitive functions. CPP correlated with disturbed visuospatial abilities in AD (r = 0.492, *p* = 0.045), indicating potential cerebral hypoperfusion and cognitive function decline in the AD group. Future research should focus on elucidating the underlying mechanisms linking vascular dysregulation, oxidative stress, and neurodegeneration in AD and NTG, aiming to develop effective interventions to prevent or slow disease progression.

## Figures and Tables

**Table 1:** Demographic and clinical characteristics of study participants.

Characteristic	AD (*N* = 17)	NTG (*N* = 35)	Controls (*N* = 46)	*p*-Value	Post-Hoc (Bonferroni)
**AGE, YEARS** **(MEAN ± SD)**	75.12 ± 4.0	73.1 ± 4.9	72.5 ± 4.9	0.169	-
**GENDER,** **MALE (%)**	8 (47.1)	8 (21.6)	12 (26.1)	0.135 (χ^2^)	-
**BMI, KG/M2** **(MEDIAN [IQR])**	26.42 [25.76–28.19]	26.70 [24.61–29.65]	27.75 [24.61–29.65]	0.843 (χ^2^)	-
**SBP, MMHG** **(MEDIAN [IQR])**	126 [118–132]	132 [124–142]	130 [125.5–136]	0.049 (χ^2^)	AD-NTG: 0.014 (0.043)
**DBP, MMHG** **(MEDIAN [IQR])**	70 [64–75]	78 [70–82]	80 [72–82]	<0.001 (χ^2^)	AD-NTG: <0.001 (0.002), AD-C: <0.001 (0.000)
**MAP, MMHG (MEDIAN [IQR])**	87.94 [82.0–94.7]	96.67 [99.0–102.3]	95.52 [91.7–100.0]	0.001 (χ^2^)	AD-NTG: <0.001 (0.002), AD-C: <0.001 (0.002)
**PP, MMHG (MEDIAN [IQR])**	56 [52–62]	52 [48–63]	51 [44–60]	0.204 (χ^2^)	-
**ICP, MMHG** **(MEAN ± SD)**	9.36 ± 2.58	8.33 ± 2.72	8.74 ± 2.89	0.445 (ANOVA)	-
**CPP, MMHG** **(MEAN ± SD)**	78.84 ± 6.33	88.6 ± 10.58	86.9 ± 7.09	<0.001 (ANOVA)	AD-NTG: <0.001 (0.003), AD-C: <0.001 (<0.001)
**BCVA (MEDIAN [IQR])**	0.85 [0.70–1.00]	0.95 [0.90–1.00]	1.0 [1.0–1.00]	<0.001 (χ^2^)	AD-C: <0.001 (0.001), NTG-C: 0.011 (0.032)
**IOP, MMHG** **(MEDIAN [IQR])**	14.00 [12.00–15.00]	14.00 [12.00–15.00]	14.00 [13.00–15.25]	0.184 (χ^2^)	-
**SE, D** **(MEDIAN [IQR])**	0.5 [−0.50–1.89]	0.38 [0.0–1.19]	0.88 [−0.19–1.75]	0.353 (χ^2^)	-
**MD, DB** **(MEDIAN [IQR])**	−4.71 [−5.74 to −2.91]	−2.86 [−5.48 to −1.55]	−0.99 [−1.83 to −0.34]	<0.001 (χ^2^)	AD-C: <0.001 (0.000), NTG-C: <0.001 (0.000)
**PSD, DB** **(MEDIAN [IQR])**	4.45 [2.73–6.51]	2.64 [1.58–4.97]	1.63 [1.37–1.89]	<0.001 (χ^2^)	AD-C: <0.001 (0.000), NTG-C: <0.001 (0.000)
**VFI, %** **(MEDIAN [IQR])**	92.00 [87.50–94.00]	95.50 [89.00–98.00]	99.00 [97.50–99.00]	<0.001 (χ^2^)	AD-C: <0.001
**OPP, MMHG** **(MEAN ± SD)**	50.28 ± 5.26	55.26 ± 6.39	54.48 ± 4.77	0.008 (ANOVA)	AD-NTG: 0.008, AD-C: 0.025
**SPP, MMHG** **(MEAN ± SD)**	110.5 ± 9.5	119.5 ± 12.7	116.4 ± 9.1	0.02 (ANOVA)	AD-NTG: 0.016
**DPP, MMHG** **(MEDIAN [IQR])**	59.0 [48.0–63.0]	65.00 [58.5–69.5]	64.4 [59.5–68.00]	0.024 (χ^2^)	AD-C: 0.008 (0.024)
**TSG, MMHG** **(MEAN ± SD)**	4.4 ± 2.5	5.58 ± 2.95	5.23 ± 2.85	0.367 (ANOVA)	-
**NORMAL BP, *N*** **(%)**	5(29.4)	12(34.3)	16(34.8)	0.901 (χ^2^)	-
**AH, *N*** **(%)**	11(64.7)	20(57.1)	28(61.0)	0.842 (χ^2^)	-
**LOW BP, *N*** **(%)**	1(5.9)	3(8.6)	2(4.4)	0.439 (χ^2^)	-
**BETA-BLOCKERS, *N*** **(%)**	7(41.2)	14(40.0)	13(28.3)	0.495 (χ^2^)	-
**ACE INHIBITORS, *N*** **(%)**	5(29.4)	8(22.9)	14(30.0)	0.707 (χ^2^)	-
**CCB, *N*** **(%)**	6(35.3)	4(11.4)	7(15.2)	0.094 (χ^2^)	-
**LIPID-LOWERING DRUGS, *N*(%)**	4(23.5)	5(14.3)	8(17.4)	0.712 (χ^2^)	-
**DIURETICS, *N*(%)**	2 (11.8)	2(6.1)	4(8.7)	0.780 (χ^2^)	-
**ANTIDEPRESSANTS, *N*(%)**	5(29.4)	3(8.6)	3(6.5)	0.014 (χ^2^)	-

ACE—Angiotensin-Converting Enzyme; AD—Alzheimer’s Disease group; AH- arterial hypertension; CCB—Calcium channel blockers; NTG—Normal-Tension Glaucoma group; C—Control group; BB—beta blockers; BP—blood pressure, BMI—Body Mass Index; SBP—Systolic Blood Pressure; DBP—Diastolic Blood Pressure; MAP—Mean Arterial Pressure; N—number; PP—Pulse Pressure; ICP—Intracranial Pressure; CPP—Cerebral Perfusion Pressure; BCVA—Best Corrected Visual Acuity; IOP—Intraocular Pressure; SE—Spherical Equivalent; MD—Mean Deviation (perimetry parameter); PSD—Pattern Standard Deviation (perimetry parameter); VFI—Visual Field Index (perimetry parameter); OPP—Ocular Perfusion Pressure; SPP—Systolic Perfusion Pressure; DPP—Diastolic Perfusion Pressure; TSG—Translaminar Pressure Gradient.

**Table 2:** Correlation between Cerebral Perfusion Pressure (CPP) and Cognitive Test Scores.

Cognitive Test		AD (r, *p*-Value)	NTG (r, *p*-Value)	Controls (r, *p*-Value)
Clock Drawing Test (CDT) scores		0.265, *p* = 0.303	0.283, *p* = 0.1	0.134, *p* = 0.382
ACE-R Subtest	Attention and Concentration	0.025, *p* = 0.925	0.164, *p* = 0.347	0.249, *p* = 0.1
	Memory	0.338, *p* = 0.185	0.079, *p* = 0.651	−0.102, *p* = 0.507
	Verbal Fluency	−0.024, *p* = 0.927	−0.138, *p* = 0.429	0.212, *p* = 0.163
	Language	0.032, *p* = 0.902	−0.034, *p* = 0.848	0.067, *p* = 0.663
	Visuospatial Abilities	0.492, *p* = 0.045	0.094, *p* = 0.592	−0.017, *p* = 0.911
	ACE-R Total Score	0.223, *p* = 0.391	0.024, *p* = 0.891	0.069, *p* = 0.650
MMSE Score		0.088, *p* = 0.737	0.112, *p* = 0.522	0.127, *p* = 0.405

**Table 3:** Correlation between Mean Arterial Pressure (MAP) and Cognitive Test Scores.

Cognitive Test		AD (r, *p*-Value)	NTG (r, *p*-Value)	Controls (r, *p*-Value)
Clock Drawing Test (CDT)		0.257, *p* = 0.320	0.378, *p* = 0.025	0.185, *p* = 0.225
ACE-R Subtest	Attention and Concentration	−0.085, *p* = 0.745	0.074, *p* = 0.674	0.213, *p* = 0.159
	Memory	0.350, *p* = 0.168	0.101, *p* = 0.564	−0.102, *p* = 0.507
	Verbal Fluency	0.128, *p* = 0.623	−0.103, *p* = 0.555	0.119, *p* = 0.437
	Language	0.055, *p* = 0.835	−0.119, *p* = 0.496	0.031, *p* = 0.837
	Visuospatial Abilities	0.365, *p* = 0.150	0.119, *p* = 0.252	−0.027, *p* = 0.862
	ACE-R Total Score	0.232, *p* = 0.371	0.022, *p* = 0.902	0.017, *p* = 0.912
MMSE Score		0.027, *p* = 0.919	0.125, *p* = 0.476	0.016, *p* = 0.915

**Table 4:** Correlations between Pulse Pressure (PP) and Cognitive Test Scores.

Cognitive Test		AD (r, *p*-Value)	NTG (r, *p*-Value)	Controls (r, *p*-Value)
Clock Drawing Test (CDT)		−0.527, *p* = 0.016	0.136, *p* = 0.435	0.074, *p* = 0.628
ACE-R Subtest	Attention and Concentration	−0.364, *p* = 0.151	−0.024, *p* = 0.890	−0.139, *p* = 0.364
	Memory	−0.018, *p* = 0.467	−0.197, *p* = 0.303	−0.232, *p* = 0.125
	Verbal Fluency	−0.416, *p* = 0.097	−0.193, *p* = 0.268	−0.440, *p* = 0.002
	Language	−0.332, *p* = 0.193	−0.053, *p* = 0.762	−0.028, *p* = 0.853
	Visuospatial Abilities	−0.373, *p* = 0.14	−0.018, *p* = 0.920	−0.063, *p* = 0.679
	ACE-R Total Score	−0.395, *p* = 0.117	−0.174, *p* = 0.316	−0.348, *p* = 0.019
MMSE Score		−0.168, *p* = 0.52	0.055, *p* = 0.756	−0.210, *p* = 0.167

## Data Availability

Due to privacy concerns and ethical considerations, access to the clinical data used in this study is restricted. The data are available upon reasonable request, subject to approval by the Regional Kaunas Biomedical Research Ethics Committee (kaunorbtek@lsmuni.lt).
